# Correlation of Clivus Length and Angle with Chronological Age, Gender, Sagittal Growth Pattern of the Jaws, and Skeletal Maturation Using Lateral Cephalometry

**DOI:** 10.30476/dentjods.2023.98275.2065

**Published:** 2024-09-01

**Authors:** Maryam Hedayatian, Saeed Azarbayejani, Alireza Omrani, Shahab Etemadi Borujeni

**Affiliations:** 1 Postgraduate Student, Dept. of Orthodontics, School of Dentistry, Islamic Azad university, Isfahan (Khorasgan) Branch, Isfahan, Iran; 2 Dept. of Orthodontics, School of Dentistry, Islamic Azad University, Isfahan (Khorasgan) Branch, Isfahan, Iran; 3 Dept. of Oral and Maxillofacial Radiology, School of Dentistry, Islamic Azad university, Isfahan (Khorasgan) Branch, Isfahan, Iran

**Keywords:** Cranial fossa, Posterior, Cervical vertebrae, Cephalometry

## Abstract

**Statement of the Problem:**

Determination of remaining growth potential of patients is the most important factor in orthodontic treatment.

**Purpose:**

This study aimed to assess the correlation of clivus length and angle with age, gender, sagittal growth pattern of the jaws, and skeletal maturation using lateral cephalometry.

**Materials and Method:**

This cross-sectional study was conducted on 390 lateral cephalograms (Vatech, paX-i3D Green, South Korea) of patients aged 6 to 25 years. The patients were assigned to three groups of skeletal class I, II, III (n=130) with equal gender distribution. The clivus length and angle, Welcher angle, maxillary and mandibular effective length, sella turcica to Nasion (SN), and the angles between SN and point A (SNA), between SN and point B (SNB), and between NA and NB (ANB )were measured. Correlations of variables with age and gender, and cervical vertebral maturation stage (CVMS) were analyzed using the Pearson and Spearman’s correlation tests, independent t-test, and one-way ANOVA at 0.05 level of significance.

**Results:**

Clivus length had a significant correlation with SNA (r= 0.103, *p*= 0.042), SNB (r= 0.108, *p*= 0.033), maxillary (r= 0.547, *p*< 0.001) and mandibular (r= 0.589, *p*< 0.001) effective lengths, SN length (r= 0.586, *p*< 0.001), and CVMS (r= 0.697, *p*< 0.001). Clivus angle had a significant correlation with SNA (r= 0.105, *p*= 0.039), SNB (r= 0.155, *p*= 0.002), maxillary (r= 0.507, *p*< 0.001) and mandibular (r= 0.596, *p*= 0.001) effective lengths, SN length (r= 0.566, *p*< 0.001), and CVMS (r= 0.699, *p*< 0.001). The mean clivus length (*p*= 0.006) and angle (*p*= 0.002) were significantly higher in males, and had a significant correlation with age (r= 0.636 and r= 0.718, *p*< 0.001). The mean clivus length and angle were not significantly different in class I, II, III (*p*> 0.05).

**Conclusion:**

All parameters were greater in males, and increased with age (except Welcher angle). Clivus length and angle had significant correlations with position of both jaws but not with sagittal relationship.

## Introduction

Dentofacial deformities are among the main reasons for patients seeking orthodontic treatment. Such patients require functional or orthopedic treatment of the jaws during their growth and development period [ 1 ]. Evidence shows that maximum response to functional and orthopedic treatments of the jaws can be achieved during the growth spurt period, because the occlusion and position of the teeth are established during this period and the changes after this period are not significant [ 1 ]. 

Since the time of puberty and developmental changes often vary in different individuals, the remaining time of growth and development and the level of skeletal maturity of patients should be necessarily determined prior to orthodontic treatment planning [ 2 ]. Skeletal maturity is determined by biological indices such as height, weight, chronological age, dental maturation, cervical vertebral maturation stage (CVMS), and development and maturity of the phalanges and the wrist bones. Among the afore-mentioned indices, hand-wrist radiography is the most reliable biological index for this purpose [ 2 ]. However, orthodontic patients already need to undergo panoramic radiography and lateral cephalometry for their orthodontic treatment planning, and these additional radiographies further expose them to radiation [ 2 ]. Thus, researchers have been in search of alternative techniques. 

Skull base has a key role in craniofacial growth and development and coordinates different growth patterns of the brain, nasal cavity, oral cavity, and the pharynx in terms of space and function [ 3 ]. Cranial base flexure is an overlooked topic in craniofacial research [ 3 ]. The clivus bone comprises the most posterior part of the skull base [ 4 ]. It has a steep surface and extends from the posterior part of the sella turcica to the foramen magnum. The sphenoid bone body forms its superior part and the clival part of the occipital bone forms its inferior part. The spheno-occipital synchondrosis is not completely ossified before the age of 18 years [ 3 - 5 ].

The significance of clivus is mainly because of the possibility of important pathologies such as chondroma, metastatic tumors, inflammation, fibrous dysplasia, and fracture in traumas. Moreover, age and sex determination according to the dimensions of clivus in forensic medicine has been the topic of some investigations [ 5 ]. 

In assessment of the dimensions of clivus, length, width and two angles related to clivus namely the clivus angle and the Welcher basal angle are often evaluated. The Welcher basal angle is the angle formed between a line extending from the planum sphenoidale and another line along the posterior border of clivus [ 6 ]. The clivus angle is formed at the intersection of a line passing along the clivus and another line passing through the posterior surface of the body of second vertebra [ 6 ]. In addition, several anatomical variations have been reported for clivus bone, which may be due to its degradation, congenital or developmental anomalies, or pathologies. Age and sex also play a role in this regard [ 7 ]. 

Lateral cephalometry is routinely requested in orthodontic treatments for assessment of the craniofacial morphology and diagnosis of dentoalveolar malocclusions, and skeletal discrepancies [ 2 ]. Evidence shows that the cervical vertebrae are a reliable index for determination of skeletal maturity [ 8 ]. However, they are not reliable for determination of termination of growth, and therefore, serial cephalograms are required for this purpose [ 9 ].

Determination of skeletal age and assessment of the remaining growth potential of patients are among the most important factors in orthodontic treatment. Thus, this study aimed to assess the correlation of clivus length and angle with chronological age, gender, sagittal growth pattern of the jaws, and skeletal maturation using lateral cephalometry. 

## Materials and Method

This cross-sectional study was conducted on the available lateral cephalograms (Vatech, paX-i3D Green, South Korea) of orthodontic patients aged 6 to 25 years retrieved from the archives of the Orthodontics Department of School of Dentistry, Islamic Azad University, Khorasgan Branch in 2021. The protocol and its ethics were approved by the Research Committee of the University (ethics code: IR.IAU.KHUISF.REC.1400.180).

### Sample size

The sample size was calculated to be 130 in each group of class I, class II, and class III patients (a total of 390) according to a previous study [ 10 ] assuming alpha= 0.05, beta=0.2, study power of 80%, and minimum correlation coefficient for a significant correlation to be 0.25. Considering 10% possible dropouts, 130 cephalograms were selected for each group. 

### Eligibility criteria

The inclusion criteria were absence of systemic and congenital diseases, absence of syndromes affecting calcification and development of bones, no previous history of surgery or trauma to the head and neck region, and no previous history of orthodontic treatment. 

### Sample selection

Lateral cephalograms were selected from the archives of the Orthodontics Department and belonged to orthodontic patients treated from 2015 to 2021. A total of 390 lateral cephalograms (130 for each class of occlusion) of patients aged 6 to 25 years (equal number of males and females, in age groups of 6-12, 13-18, and 19-25 years) were selected by convenience sampling. 

### Measurement of variables

**Figure 1:**
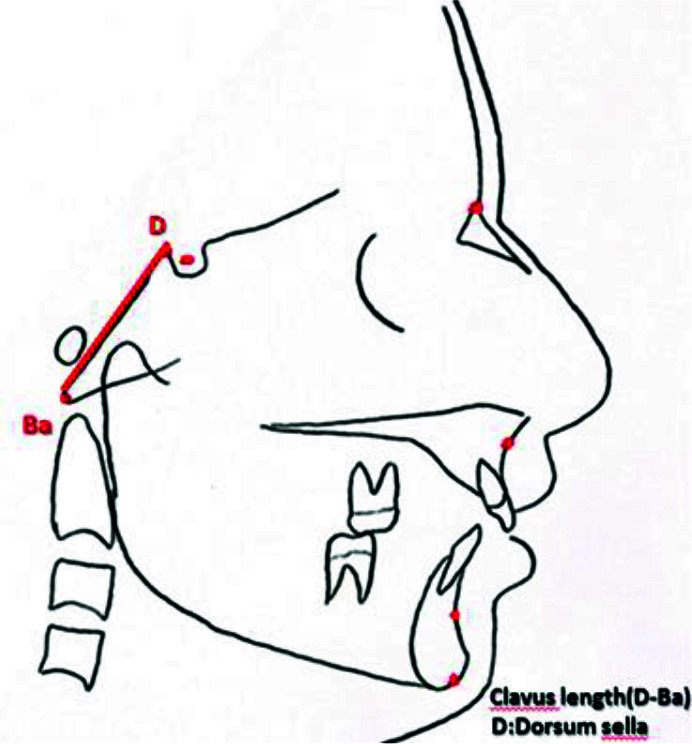
Clivus length (dorsum sella to basion)

**Figure 2:**
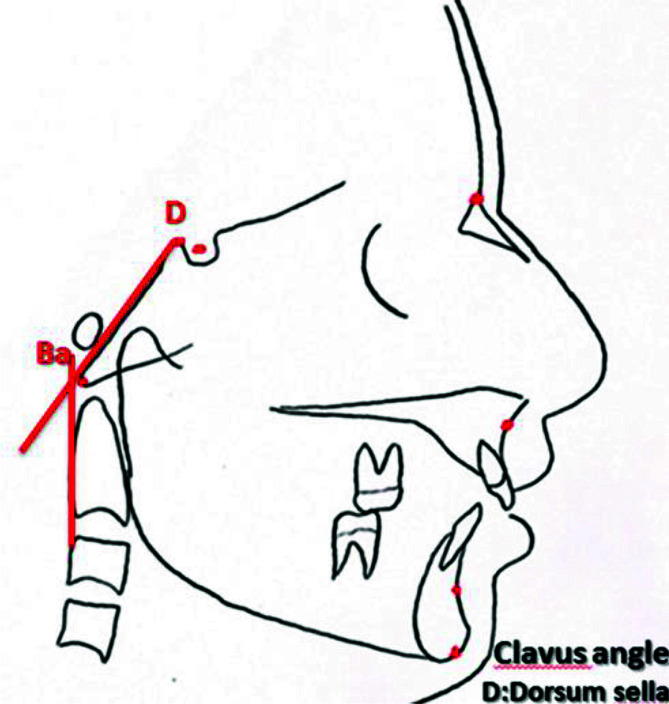
Clivus angle; intersection of the line along the posterior surface of the clivus with the line that passes through the posterior extension of the trunk of the neck vertebrae

**Figure 3:**
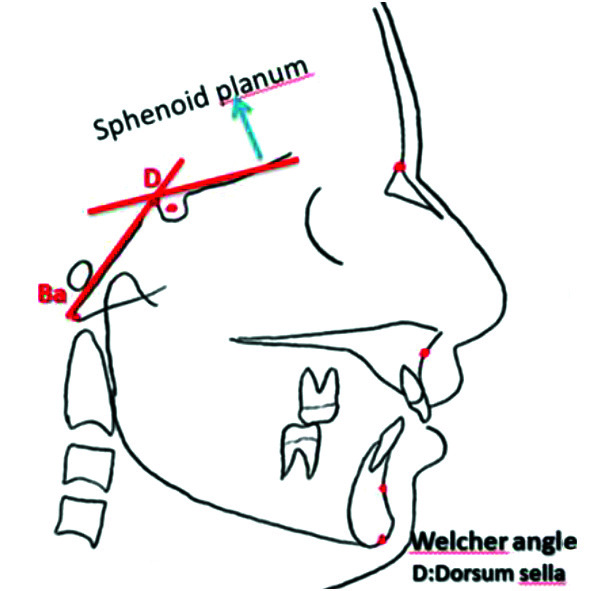
Welcher angle; The intersection of the line that passes through the sphenoid plenum and the clivus

**Figure 4:**
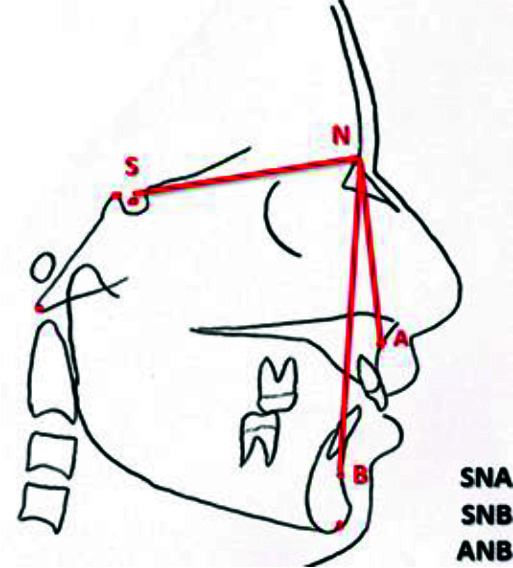
Length of SN line and SNA, SNA, ANB angles

**Figure 5:**
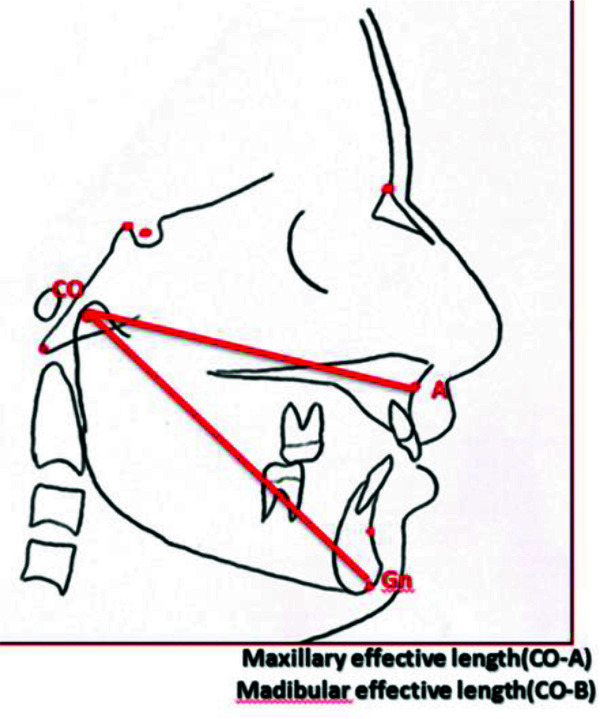
Maxillary effective length (CO-A) and Mandibular effective length (CO-B)

**Figure 6:**
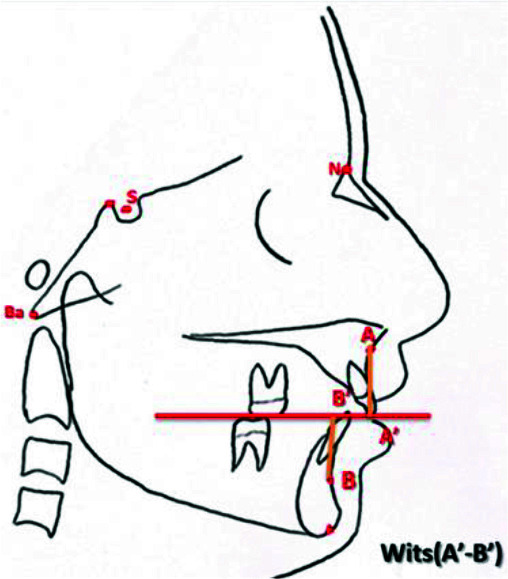
Wits appraisal (A’-B’)

**Figure 7:**
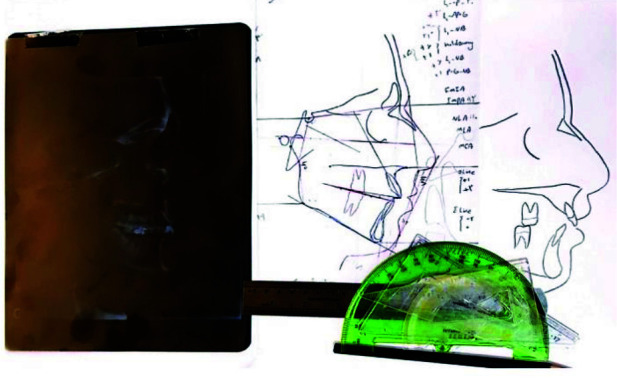
Tracing cephalograms

**Figure 8:**
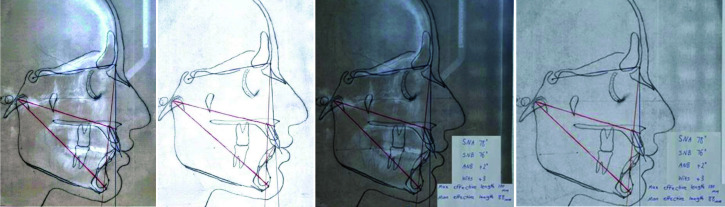
Tracing cephalograms (SNA,SNB,ANB,Wits appraisal,maxillary effective length and mandibular effective length)

**Figure 9:**
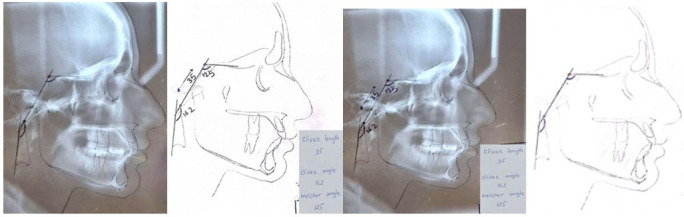
Tracing cephalograms (clivus length, clivus angle,welcher angle)

The lateral cephalograms (Vatech, paX-i3D Green, South Korea) were manually traced using matte acetate tracing papers with 0.75mm thickness (8x10 inch) and a HB pencil with a sharp tip (Figure 1). The length and angle of clivus (Figures 2-3), Welcher angle (Figure 4), the length of sella turcica (S) to Nasion (N) (SN) line (Figure 5), the angle between SN plan and the deepest point on the curvature of the maxillary alveolar process (point A) (SNA), the angle between SN plan and the deepest point on the curvature of the mandibular alveolar process (point B) (SNB), and the angle between NA and NB plan (ANB) (Figure 5), the maxillary and mandibular effective length (Figure 6), the Wits appraisal (the li-near distance between the points of contact of the perpendiculars on the occlusal plane, AO and BO, indicated the skeletal sagittal jaw relationship) (Figure 7), were all measured manually and also by using a protractor (Figures 8-9).

### Assessment of CVMS

The CVMS was determined by assessing the cervical vertebrae and based on the presence of concavity in the 

lower border of the body of C2, C3 and C4 and the shape of the body of C3 and C4. Accordingly, the CVMS (Table 1) was determined using the following classification [ 11 ].

**Table 1:** CVMS(cervical vertebral maturation stage)

CVMS I	The inferior border of all three vertebrae is smooth.
CVMS II	The inferior border of C2 is concave and the body of C3 and C4 is trapezoidal-shaped.
CVMS III	The inferior border of all three vertebrae is concave and the body of C3 and/or C4 has a trapezoidal shape.
CVMS IV	The inferior border of C2, C3 and C4 is concave, and the body of both C3 and C4 has a horizontal rectangular shape.
CVMS V	The inferior border of C2, C3, and C4 is concave, and the body of C3 or C4 is square-shaped.
CVMS VI	The inferior border of C2, C3, and C4 is concave, and the body of at least C3 or C4 has a vertical rectangular shape.

### Determination of sagittal skeletal pattern according to the Steiner analysis (skeletal class I, II, and III)

The SN reference line was first drawn, and the SNA angle was measured. If the SNA angle was 82±2 degrees, the position of the maxilla was considered to be normal. If the SNA angle was < 80 degrees, the patient was diagnosed with maxillary retrognathism. If the SNA angle was > 84 degrees, the patient was diagnosed with maxillary prognathism.

The SNB angle was then measured. If the SNB angle was 80±2 degrees, the mandible had a normal position. If the SNB angle was < 78 degrees, the patient was diagnosed with mandibular retrognathism. If the SNB angle was > 82 degrees, the patient was diagnosed with mandibular prognathism.

The ANB angle was also measured. If the ANB angle was 2±2 degrees, the patient was skeletal class I. If the ANB angle was < 0 degrees, the patient was diagnosed to be skeletal class III. If the ANB angle was > 4 degrees, the patient was diagnosed to be skeletal class II. 

### Clivus measurements

The length of clivus was measured as the longest superior-inferior distance between the superior point of the dorsum sella and the inferior point of the anterior margin of foramen magnum, and the mean value was calculated for all three age groups, males and females, each class of occlusion, and all 6 CVMSs [ 5 ].

The clivus angle was measured at the intersection of a line along the posterior border of clivus and another line passing from the posterior border of cervical vertebrae, and the mean of the value was calculated and reported for all three age groups, males and females, each class of occlusion, and all 6 CVMSs [ 6 ]. 

The Welcher angle was also measured at the intersection of a line extending from the planum sphenoidale and another line from the clivus, and the mean of the value was calculated and reported for all three age groups, males and females, each class of occlusion, and all 6 CVMSs [ 6 ]. Finally, the correlation of length and angle of clivus with age, gender, sagittal skeletal growth pattern, and CVMS was analyzed. 

### Statistical analysis

Data were analyzed by SPSS version 24 using the Pearson and Spearman’s correlation tests, independent t-test, and one-way ANOVA at 0.05 level of significance. 

## Results

A total of 390 lateral cephalograms of 195 males (50%) and 195 females (50%) were evaluated. The majority of participants were 11 to 15 years old (34.9%), and the mean age of participants was 15.91±4.96 years.

Of a total of 390 lateral cephalograms, 130 (33.3%) belonged to skeletal class I, 130 (33.3%) belonged to skeletal class II, and 130 (33.3%) belonged to skeletal class III participants. Table 2 presents the frequency of CVMS in the study population. 

**Table 2:** Frequency of cervical vertebral maturation stage (CVMS) in the study population

CVMS	Number	Frequency
I	34	8.7
II	34	8.7
III	28	7.2
IV	56	14.4
V	60	15.4
VI	178	45.6
Total	390	100.0

Table 3 presents the measures of central dispersion for the clivus length and angle, Welcher angle, SNA, SNB, and ANB angles, maxillary and mandibular effective length, SN length, and Wits appraisal in the study population as measured on lateral cephalograms.

**Table 3:** Measures of central dispersion for the clivus length and angle, Welcher angle, SNA, SNB, and ANB angles, maxillary and mandibular effective length, SN length, and Wits appraisal in the study population (n=390)

Variable	Minimum	Maximum	Mean	Std. deviation
Clivus length	30.00	50.00	42.91	3.41
Clivus angle	135.00	172.00	152.15	8.43
Welcher angle	102.00	131.00	117.89	7.06
SNA	71.00	87.00	79.70	2.87
SNB	69.00	83.00	77.50	2.85
ANB	-5.00	9.00	2.15	3.37
Maxillary effective length	65.00	106.00	87.02	8.01
Mandibular effective length	86.00	140.00	111.73	11.07
SN length	50.00	79.00	67.63	6.14
Wits appraisal	-5.00	5.00	-.14	2.72

The Pearson’s correlation test revealed significant correlations (from the lowest to the highest) between the clivus length and SNA (r= 0.103, *p*= 0.042), SNB (r= 0.108, *p*= 0.033), maxillary effective length (r= 0.547, *p*< 0.001), mandibular effective length (r= 0.589, *p*< 0.001), and SN length (r= 0.586, *p*< 0.001). In addition, the Spearman’s correlation test showed a significant correlation between the clivus length and CVMS (r= 0.697, *p*< 0.001). The Pearson’s correlation test found no significant correlation between the clivus length and ANB (r= -0.015, *p*= 0.765) or Wits appraisal (r= 0.056, *p*= 0.271).

The Pearson’s correlation test found significant correlations (from the lowest to the highest) between the clivus angle and SNA (r= 0.105, *p*= 0.039), SNB (r= 0.155, *p*= 0.002), maxillary effective length (r=0.507, *p*< 0.001), mandibular effective length (r= 0.596, *p*= 0.001), and SN length (r= 0.566, *p*< 0.001). The Spearman’s correlation test showed a significant correlation between the clivus angle and CVMS (r= 0.699, *p*< 0.001). The Pearson’s correlation test revealed no significant correlation between the clivus angle and ANB (r= -0.039, *p*= 0.439) or Wits appraisal (r= -0.044, *p*= 0.388). 

The Pearson’s correlation test showed significant correlations (from the lowest to the highest) between the Welcher angle and SNA (r= 0.196, *p*< 0.001), SNB (r= 0.103, *p*= 0.043), Wits appraisal (r= 0.113, *p*= 0.026), maxillary effective length (r= 0.612, *p*< 0.001), mandibular effective length (r= 0.626, *p*< 0.001), and SN length (r= 0.667, *p*< 0.001). The Spearman’s correlation test also revealed a significant correlation between the Welcher angle and CVMS (r= 0.682, *p*< 0.001). The Pearson’s correlation test found no significant correlation between the Welcher angle and ANB (r= 0.084, *p*= 0.099). 

Table 4 compares the clivus length according to gender, age, and skeletal class of participants. Independent t-test showed significantly higher clivus length in males than females (*p*= 0.006). The Pearson’s correlation test found a significant correlation between the clivus length and age (r= 0.636, *p*< 0.001). One-way ANOVA indicated a significant difference in the mean clivus length among the three skeletal classes (*p*= 0.018) such that the mean clivus length in skeletal class I individuals was significantly higher than that in class II and class III individuals.

**Table 4:** Comparison of the clivus length according to gender, age, and skeletal class of participants

Variable	Category	Number	Mean	Std. deviation	Statistic	*p* Value
Gender	Females	195	42.44	3.40	t=-2.741	.006
	Males	195	43.38	3.36		
Age (yrs.)	< 10	54	39.09	2.98	<.636^**^	<.001
	11-15	136	41.44	2.80		
	16-20	128	44.97	2.42		
	21-25	72	44.89	2.20		
Skeletal class	I	130	42.22	3.46	F=4.041	.018
	II	130	43.29	3.32		
	III	130	43.22	3.36		

Table 5 compares the clivus angle according to gender, age, and skeletal class of participants. The mean clivus angle in males was significantly larger than that in females (*p*= 0.002). The Pearson’s correlation test showed a significant correlation between the clivus angle and age (r= 0.718, *p*< 0.001). One-way ANOVA revealed no significant correlation in clivus angle among class I, II, and III individuals (*p*= 0.142). 

**Table 5:** Comparison of the clivus angle according to gender, age, and skeletal class of participants

Variable	Category	Number	Mean	Std. deviation	Statistic	*p* value
Gender	Females	195	150.80	8.54	t=-3.192	.002
	Males	195	153.49	8.11		
Age (yrs.)	< 10	54	143.91	4.09	<.718^**^	<.001
	11-15	136	147.58	6.62		
	16-20	128	155.96	6.83		
	21-25	72	160.17	5.18		
Skeletal class	I	130	152.96	7.98	F=1.963	.142
	II	130	150.98	9.41		
	III	130	152.49	7.72		

Table 6 compares the Welcher angle according to gender, age, and skeletal class of participants. Independent t-test showed that the mean Welcher angle in males was significantly larger than that in females (*p*= 0.002). The Pearson’s correlation test showed a significant inverse correlation between the Welcher angle and age (r= 0.710, *p*< 0.001). One-way ANOVA found no significant difference in the mean Welcher angle among class I, II, and III individuals (*p*= 0.288). 

**Table 6:** Comparison of the Welcher angle according to gender, age, and skeletal class of participants

Variable	Category	Number	Mean	Std. deviation	Statistic	*p* value
Gender	Females	195	116.81	7.26	t=-3.081	.002
	Males	195	118.98	6.70		
Age	< 10	54	123.57	4.27	<.710^**^	<.001
	11-15	136	121.30	6.76		
	16-20	128	114.85	4.61		
	21-25	72	109.93	3.87		
Skeletal class	I	130	117.68	8.50	F=1.249	.288
	II	130	118.67	5.97		
	III	130	117.34	6.45		

The results showed that 50% of the changes in the clivus length were determined by the effect of CVMS, mandibular effective length, skeletal class of occlusion, age, and gender (*p*< 0.001 for all).

CVMS had the greatest share in determination of the clivus length followed by age, and mandibular effective length. Per each one unit increase in CVMS, the clivus length increased by averagely 0.565mm. Per each 1 year increase in age, the clivus length increased by 0.162mm. Per each 1 unit increase in mandibular effective length, the clivus length averagely increased by 0.072 mm. 

Regression analysis showed that 55% of the changes in the clivus angle were determined by the effect of CVMS, age, and gender (*p*< 0.001 for all). 

Age had the greatest share in determination of the clivus angle followed by CVMS and gender. Each 1 year increase in age increased the clivus angle by averagely 0.933 degrees. Each 1 unit increase in CVMS increased the clivus angle by averagely 2.7 degrees. Also, the clivus angle in males was larger than that in females by averagely 1 degree. 

The results showed that 58% of the changes in the Welcher angle were determined by the effect of age, SN length, gender, and CVMS (*p*< 0.001). 

Age had the greatest share in determination of the Welcher angle followed by SN length, CVMS, and gender. Per each 1 year increase in age, the Welcher angle averagely decreased for 0.568 degrees. Per each 1 unit increase in SN length, the Welcher angle averagely increased by 0.294 degrees. Per each 1 unit increase in CVMS, the Welcher angle averagely decreased for 0.663 degrees. The Welcher angle in males was averagely 1.68 degrees larger than that in females. 

## Discussion

This study assessed the correlation of clivus length and angle with chronological age, gender, sagittal growth pattern of the jaws, and skeletal maturation using lateral cephalometry. 

Spheno-occipital synchondrosis is often closed at around 16-17 years in females and 18-19 years in males [ 12 ]. Radiographically, the spheno-occipital synchondrosis shows active growth by 10 to 13 years of age. At this age, it starts to close from the superior towards the inferior region and continues by 11 to 14 years of age in females and 13 to 16 years in males [ 12 ]. In the present study, the mean clivus length in our study population with a mean age of 15.91±4.96 years was 42.44±3.40 mm in females and 43.38±3.36 mm in males. There was a significant relationship between the length of the clivus bone and gender. The average length of clivus bone in boys was significantly higher than that in girls.

This is due to faster growth rate, stronger growth spurt, and longer growth period in boys [ 12 ].

There was a direct and significant relationship between the length of the clivus bone and age. These values were 44 mm in females and 45.85 mm in males in a study by Bayrak and Bulut [ 13 ] on a population with a mean age of 17±10.8 years. They also reported a direct correlation between the clivus length with age and gender [ 13 ].Monirifard *et al*. [ 14 ] showed that all dimensions of the cranial base were larger in males than females. Chaurasia *et al*. [ 5 ] reported the mean clivus length to be 45.53 mm in males and 43.1 mm in females, in a population between 6 to 78 years of age. They also reported direct correlation of the clivus length with age and gender [ 5 ]. The above mentioned studies [ 5 , 13 - 14 ] confirmed the present results regarding significantly higher clivus length in males than females. The present results also revealed significant correlation of clivus length with age which was in line with the previous literature [ 5 , 13 ]. The mean clivus length in a study by Joaquim *et al*. [ 6 ] was found to be 38mm the mean value reported in the present study probably due to different age range of participants. Henneberke and Prahl [ 15 ] showed that the greatest change in linear dimensions of the cranial base occurs between 6-15 years at the post-sphenoid region, with a mean rate of 1 mm/year in males and 0.9 mm/year in females. Thus, the mean length of the posterior skull base in males is 2.5 mm larger than that in females. The present analyses showed that per each 1 year increase in age (6 to 25 years), the clivus length averagely increased by 0.162 mm, which was much lower than the rate reported in previous studies [ 6 , 15 ]. This difference may be due to age and racial differences of the study populations. 

There is a direct and significant relationship between the length of the clivus bone and age. In previous studies [ 12 , 15 ], this increase in length was significant up to 14,15 years old, but it was shown that until late adulthood, a slight increase in length also occurs. The increase in the length of the posterior base of the skull until the age of 16 is mainly due to the growth of the spheno-occipital synchondres, and after that, it continues to grow and increase in length with surface deposition (modeling) [ 12 ].

The present results showed a significant correlation between the clivus length and CVMS. Per each 1 unit increase in CVMS, the clivus length averagely increased by 0.565mm. Melta *et al*. [ 16 ] found that the greatest change in the posterior cranial base occurs between CS3/4 and CS1/2 stages. 

Considering that the length of the clivus was related to the more anterior position of the maxilla and mandible, it seems logical that it does not affect the Wits appraisal and ANB. The present study also revealed a significant correlation between the clivus length and SN (length of the anterior skull base) such that individuals with a shorter anterior skull base also had a shorter clivus and vice versa. This is because the growth of the anterior base of the skull might affect the growth of other craniofacial components [ 12 ].

 Moreover, the clivus length had a significant correlation with SNA and maxillary effective length such that longer clivus was associated with longer maxilla and its more forward position. Monirifard *et al*. [ 14 ] indicated significant correlation of the maxillary effective length with anterior cranial base length, posterior cranial base length, and the overall length of the cranial base, which was in line with the present results. However, unlike the present study, they found a significant inverse correlation between the posterior cranial base length and SNA, which may be due to differences in the anatomical landmark of S-Ba in their study population and clivus dimensions as well as the sample size and age range of the participants in the two studies. The length of the base of the skull is related to the position of the maxilla (the longer it is, the more anterior the maxilla is), so logically, the clivus length has a direct relationship with the position of the maxilla [ 12 ].

The present results showed significant correlations between the clivus length, SNB, and mandibular effective length, which were consistent with the findings of Monirifard *et al*. [ 14 ] who showed that the mandibular effective length had a direct correlation with the anterior, posterior, and overall cranial base length. A cephalometric study indicated that the posterior cranial base, due to its vicinity to the mandible, has a more prominent role in class III skeletal development. The glenoid fossa is located in the posterior cranial base and any increase in the posterior cranial base dimension results in backward shift of the glenoid fossa and subsequently the mandible [ 3 , 12 ]. Andria *et al*. [ 17 ] demonstrated that Ba-S length had a significant inverse correlation with the facial angle and point B, which was in contrast to the results of previous studies[ 12 , 14 ], and can be attributed to racial differences and counterpart compensation. The present results showed an increase in mandibular effective length and its more anterior positioning by an increase in clivus length, each 1 unit increase in the clivus length increased the mandibular effective length by averagely 0.072 mm. Maybe the reason for the more anterior position of the mandible in our study is related to the compensations of the counterparts [ 12 ]. 

The present results found no significant correlation between the clivus length and ANB angle or Wits appraisal. In addition, the mean clivus length was not significantly different in class I, II, and III individuals. These results are in contrast to the findings of previous studies [ 14 , 18 - 22 ], which may be due to differences in landmarks related to the posterior skull base and clivus, and counterpart compensation that can alter the maxillomandibular relationship. Considering that the effect of increasing the length of the clivus on the maxilla and mandible is the same, it seems logical that it does not affect the Wits appraisal and ANB [ 12 ].

The present findings showed that the mean clivus angle in males was significantly larger than that in females by averagely 1 degree. Moreover, the mean Welcher angle in males was significantly larger than that in females by averagely 1.68 degrees. In general, the mean of all cranial base-related parameters (linear and angular) was significantly larger in males than females. Graber *et al*. [ 12 ] showed that gender differences in cranial base are due to faster growth in males and their growth and development occurs over a longer period of time [ 12 ]. Also, another study [ 23 ] found that gender differences in cranial base-related parameters (linear and angular) were significantly more common in class I patients such that all these parameters were greater in males (except for N-S-Ba); this result was different from the present findings. This controversy may be due to racial differences between the two study populations. They found no gender-related differences in cranial base parameters in class II and III individuals [ 23 ]. 

The present study found a significant correlation between the clivus angle and age, such that per each 1-year increase in age, the clivus angle increased by approximately 0.933 degrees. 

The clivus angle is different from the cranial base angle, and can be affected by the position of the head and cervical vertebrae. Joaquim *et al*. [ 6 ] reported the mean clivus angle to be 154.9 degrees in individuals over 18 years of age, which was in line with the present results. The clivus angle was 148.42±9.88 degrees in normal individuals in a study by Botelho and Ferreira [ 24 ] and 144.5 degrees in a study by Martin *et al*. [ 25 ] in individuals with a mean age of 10.4 years.

 The increase of the clivus angle with age can be related to the growth of the surrounding soft tissues, especially changes in the airway (functional matrix theory) [ 12 ].

In the present study, the Welcher angle had a significant inverse correlation with age, and per each 1 year increase in age, the Welcher angle decreased by 0.568 degrees. The mean Welcher angle was 112.5 degrees in individuals over 18 years in the study by Joaquim *et al*, [ 6 ] using computed tomography. The mean Welcher angle was 115 degrees in adults and 114.7 degrees in children in a study by Hirunpat *et al*. [ 26 ] using magnetic resonance imaging. They suggested that these values could serve as a standard reference for assessment of skull growth in Southeast Asia but found no significant difference in size of the Welcher angle between children and adults. Krishnaswamy *et al*. [ 30 ] using cone beam computed tomographic images (CBCT) and reported a constant dynamic change in the value of the parameter planum clival angle that progresses until completion of spheno-occipital synchondrosis fusion. Differences in the reported values can be explained by the differences in age range, sample size, and race. 

The clivus angle had a significant correlation with CVMS such that by each 1 unit increase in CVMS, the clivus angle averagely increased by 2.7 degrees. The Welcher angle had a significant inverse correlation with CVMS such that by each one unit increase in CVMS, the Welcher angle averagely decreased by 0.663 degrees. Thus, aging increases the clivus angle and decreases the Welcher angle. 

Considering that with increasing age, differential remodeling at the base of the skull (more growth on the upper side) leads to a decrease in the cranial base angle, decreasing the Welcher angle seems logical [ 12 ].

The clivus angle and Welcher angle also had a significant correlation with SN length and per each 1-unit increase in SN length, the Welcher angle averagely decreased by 0.294 degrees. Thus, individuals with a larger anterior skull base have a larger clivus angle and smaller Welcher angle. The reason can be the compensation of counterparts [ 12 ].

 In addition, the clivus angle and Welcher angle had a significant correlation with SNA, and maxillary effective length. This result was in contrast to the findings of Järvinen[ 27 ] and Klock *et al*. [ 28 ] who showed that by an increase in cranial base angle, SNA decreased and vice versa, and Andria *et al*. [ 17 ] who found no significant correlation between the cranial base angle and SNA. Controversy in the results can be due to differences in anatomical landmarks and variations in study populations. However, consistent with the present findings, Graber *et al*. [ 12 ] confirmed the correlation of cranial base angle and SNA showing larger clivus and Welcher angles in individuals with maxillary prognathism. The longer the base of the skull and the larger the angle of the cranial base is, the more anterior the position of the maxilla is, so the results of our study seem reasonable [ 12 ]. 

Clivus angle and Welcher angle had a significant correlation with SNB and mandibular effective length. Monirifard *et al*. [ 14 ] and Andria *et al*. [ 17 ] found no significant correlation between the cranial base angle and SNB. Due to the effect of the position of the posterior cranial base on the location of the glenoid fossa, the existence of a relationship between the cranial base and the position of the mandible seems logical [ 12 ]. Difference between their results and the present findings can be due to differences in clivus landmark, race, or counterpart compensation. 

The Welcher angle was not significantly different among class I, II, and III individuals in the present study. A previous study showed that the skull base angle in class II division 1 patients was larger than normal occlusion and class I patients but found no difference between class I malocclusion and class II division 2 patients [ 29 ]. An acute cranial base angle results in more anterior positioning of the mandible and class III malocclusion [ 3 ]. The skull base angle alone cannot determine malocclusion. In the study by Monirifard *et al*. [ 14 ], the cranial base angle in class III patients was significantly smaller than class II patients. Similar results were reported by Sanborn *et al*. [ 29 ]. Considering the fact that the Welcher angle change has a similar effect on the position of the maxilla and mandible, it seems logical that it does not affect the Wits appraisal and ANB [ 12 ]. Controversy in the results on this topic can be due to racial and sample size differences, and counterpart compensation. 

Use of lateral cephalometry was a limitation of this study due to its two-dimensional nature. Also, absence of follow-up cephalograms of patients for assessment of the changes of clivus over time in the same patients was another limitation. Longitudinal studies are required to address this topic. Future studies are recommended to use three-dimensional imaging modalities for more accurate assessments. In addition, the distal end of clivus and its changes in different age groups and genders should be further investigated. The correlation of clivus angle with head position and respiration of patients and its possible correlation with sleep apnea are among other interesting topics for further research in this field. 

## Conclusion

All clivus-related parameters were greater in males than females, and increased with age (except for the Welcher angle), and may be used to assess growth and development. The clivus length and angle had a significant correlation with position and length of both jaws and length of anterior skull base but not with sagittal relationship of the jaws.
